# Emotional Processing and Its Association to Somatic Symptom Change in Emotional Awareness and Expression Therapy for Somatic Symptom Disorder: A Preliminary Mediation Investigation

**DOI:** 10.3389/fpsyg.2021.712518

**Published:** 2021-10-07

**Authors:** Daniel Maroti, Brjánn Ljótsson, Mark A. Lumley, Howard Schubiner, Henrik Hallberg, Per-Åke Olsson, Robert Johansson

**Affiliations:** ^1^Division of Psychology, Department of Clinical Neuroscience, Karolinska Institutet, Stockholm, Sweden; ^2^Department of Psychology, Wayne State University, Detroit, MI, United States; ^3^Department of Internal Medicine, Ascension Providence-Providence Hospital, Michigan State University College of Human Medicine, Southfield, MI, United States; ^4^Department of Psychology, Stockholm University, Stockholm, Sweden

**Keywords:** emotional awareness and expression therapy, emotional processing, Emotional Processing Scale, functional syndromes, mediation analysis, somatic symptom disorder

## Abstract

**Objective:** The aim of this study was to investigate emotional processing as a potential mediator in therapist-guided, internet-based Emotional Awareness and Expression Therapy (I-EAET) for somatic symptom disorder, using data from a previously published pilot study.

**Methods:** Participants (*N* = 52) engaged in a 9-week I-EAET treatment. Before treatment and each week during treatment (i.e., 10 weekly measurements), emotional processing was assessed with the Emotional Processing Scale-25 (EPS-25), which contains five subscales, and somatic symptoms were assessed with the Patient Health Questionnaire-15 (PHQ-15).

**Results:** Mediation analyses using linear mixed models showed that two EPS-25 subscales—Signs of Unprocessed Emotions and Impoverished Emotional Experience—were uniquely associated with somatic symptom reduction. The proportion of the mediated effect was 0.49, indicating that about half of the total association of the PHQ-15 with symptoms was accounted for by the two EPS-25 subscales.

**Conclusion:** This preliminary mediation analysis suggests that improved emotional processing is associated with change in somatic symptoms in I-EAET. However, randomized controlled and comparison trials are needed to establish that I-EAET creates the change in emotional processing and that such changes are specific to I-EAET.

## Introduction

Emotional Awareness and Expression Therapy (EAET) is a newly developed therapy for patients with chronic somatic symptoms stemming from central sensitization or amplification (Lumley and Schubiner, 2019). EAET, which integrates short-term psychodynamic therapy, emotion-focused therapy, and exposure therapy, proposes that addressing the consequences of trauma or stressful life events by increasing emotional awareness and engaging in emotional processing reduces patients’ symptoms. EAET has been found to be superior to treatment as usual, education controls, or even CBT in randomized controlled trials in patients with fibromyalgia (Lumley et al., 2017), irritable bowel syndrome (IBS) (Thakur et al., 2017), pelvic pain (Carty et al., 2019), medically unexplained symptoms (Ziadni et al., 2018), and musculoskeletal pain (Yarns et al., 2020).

We have developed an internet-administrated version of EAET (I-EAET) that is self-guided but with therapist support (Maroti et al., 2021). I-EAET includes four components: (a) pain neuroscience psychoeducation to help patients reattribute symptoms to central nervous system processes; (b) the identification of possible connections between stressful life events and somatic symptoms; (c) anxiety regulation via daily self-compassion meditations; and (d) emotional exposure and processing using expressive writing and being more expressive and assertive in relationships. The emotional exposure component, which targets the processing of suppressed or avoided emotions, is thought to be the key component leading to somatic symptom reduction.

In an uncontrolled pilot trial (Maroti et al., 2021), 52 participants with somatic symptom disorder concurrent with central sensitization engaged in 9 weeks of I-EAET, which included weekly contact with an online therapist, who gave feedback on homework assignments. Within-treatment effect sizes were large for somatic symptom reduction at both post treatment and at 4-month follow up, and the majority of patients (71.2%) achieved at least a minimally clinically significant change in somatic symptoms.

Despite EAET’s effectiveness, little is known about the mechanisms by which EAET achieve its effects. In theory, emotional processing is a key mechanism (Lumley and Schubiner, 2019), as deficits in emotional processing have been identified in patients with chronic pain and IBS (Baker et al., 2010; Esteves et al., 2013; Phillips et al., 2013; Gay et al., 2019), and problems in emotional processing have been found to mediate the association between childhood adversity and the development of psychiatric (Chung and Chen, 2017) and somatic symptoms (Mozhgan et al., 2020). Facets of emotional processing, such as emotional differentiation, naming, experiencing, tolerating, and expression, are believed to be a core mechanism in psychodynamic treatments of certain conditions (Messer, 2013; Høglend and Hagtvet, 2019). For example, in a study of panic-focused psychodynamic treatment, expressions of sadness/grief lead to a reduction of panic symptoms (Keefe et al., 2019).

To investigate emotional processing in our pilot trial of I-EAET, we assessed changes in emotional processing and somatic symptoms before treatment and weekly during treatment. In this paper, we examined whether an increased capacity for emotional processing is related to reduced somatic symptoms during and following I-EAET.

## Materials and Methods

### Participants

The sample consisted of 52 participants (96.2% female; mean age of 49.6 years) with somatic symptom disorder with centralized symptoms who self-referred for the trial. The most common somatic condition reported by patients was fibromyalgia (42.3% of patients). The sample had substantial psychiatric comorbidity, with over 80% of the participants having a psychiatric diagnosis. Nearly a third of the patients were on sick leave (30.8%), and two-thirds (*n* = 35) had ongoing pharmacological treatment. A detailed description, including inclusion and exclusion criteria and treatment content is found in Maroti et al. (2021).

### Measures

#### The Emotional Processing Scale (EPS-25)

Baker et al. (2010) and Gay et al. (2019) measures five facets of emotional processing (Impoverished Emotional Experience, Signs of Unprocessed Emotion, Avoidance, Suppression, and Unregulated Emotion). Items are rated from 0 (*completely disagree*) to 9 (*completely agree*) and averaged for each subscale. Lower scores indicate less difficulties on each facet of emotional processing. The EPS-25 subscales were analyzed as putative mediators in the present study.

The Emotional Processing Scale (EPS) was used to assess emotional processing. This scale has been validated in several studies, is widely used and has been translated to 13 languages (Baker et al., 2010; Orbegozo et al., 2018; Lauriola et al., 2021). It has been found to be sensitive to change following treatment (Baker et al., 2012; Williams et al., 2018).

#### The Patient Health Questionnaire-15

To investigate somatic symptoms, the Patient Health Questionaire-15 was employed. The PHQ-15 consists of 15 somatic symptoms that patients’ rate *not bothered at all* (0), *bothered a little* (1), or *bothered a lot* (2); ratings are summed for a total score. The PHQ-15 was pre-defined as the trial’s primary outcome. PHQ-15 is a well validated questionnaire (Kroenke et al., 2010) with fair to good psychometric properties in a Swedish population (Nordin et al., 2013) and has been found to be a moderately reliable questionnaire for the detection of somatic symptom disorder in the general population (Laferton et al., 2017). Moreover, PHQ-15 can adequately capture disease severity in patients with Fibromyalgia (Häuser et al., 2014) a condition with quite a substantial overlap with SSD (Axelsson et al., 2020). PHQ-15 have also been used as an indicator of treatment effect in several studies (Kroenke et al., 2006; Haggarty et al., 2016).

The instruments were administrated before the 9-week treatment and weekly during the treatment; that is, the dataset included 10 weekly measurements of both measures.

### Statistical Analyses

Mediation analysis investigates the extent to the effect of a predictor variable on an outcome variable (usually treatment effect) is explained by the effect of predictor variable on a third variable, the mediator, which in turn affects the outcome. In the context of the data collected in the present study, the predictor in a mediation analysis is time or week; that is, we expected there to be an effect of treatment week on the outcome variable PHQ-15. Similarly, we expected that there would be an effect of time on the EPS subscales. Finally, we expected that over the 10 assessment points, there would be a relationship between the PHQ-15 and the EPS subscales. The aim of this mediation analysis, therefore, was to determine how much of the per-week improvement on the outcome (PHQ-15) was explained by change in the mediators (EPS-subscales) (Baron and Kenny, 1986; Preacher and Hayes, 2008). A stepwise mediation analysis was used. First, we determined the rate of weekly improvement on the outcome, PHQ-15, during the treatment (i.e., the *c*-path). Second, the association between treatment week and each of the mediators (i.e., one *a*-path for each mediator) was investigated. Third, the relationship between each mediator and the PHQ-15 (i.e., *b*-path) throughout the treatment period was estimated, controlling for treatment week. This third step was initially performed separately for each mediator by itself as a single mediator analysis and then with all mediators together to form a multiple mediator analysis (Preacher and Hayes, 2008), to investigate each mediator’s unique contribution to improvement in somatic symptoms. Lastly, the *a* and *b*-path estimate for each mediator (from the single and multiple mediator analyses) were multiplied to form *ab*-products, which is the indirect, or mediated effect (i.e., how much of the effect of treatment week on the outcome that is explained by change in the mediator). We also calculated the proportion of the total effect that was accounted for by the mediators, using the formula ab/c (Preacher and Kelley, 2011).

All analyses were performed in R (R Core Team, 2021) and used linear mixed models with random intercept to account for dependency between the weekly measurements. To determine confidence intervals for the indirect effects (the ab-products), 5000 bootstrap replications of all analyses were conducted. Statistically significant mediation meant that the confidence intervals did not contain zero (Preacher and Hayes, 2008).

## Results

Table 1 depicts observed means, standard deviations, and number of observations for outcome and processes over the treatment period. Both PHQ-15 and EPS-25 showed a decreasing trend during treatment, implying a reduction in both somatic symptoms and emotional processing difficulties.

**TABLE 1:** Observed means, standard deviations, and number of observations for outcome and processes over the treatment period.

Week	0	1	2	3	4	5	6	7	8	9
N	48	52	51	50	46	46	45	45	45	52
PHQ-15 Mean (SD)	13.77 (3.68)	13.4 (3.77)	13.47 (3.91)	12.38 (3.83)	11.76 (4.71)	12.45 (3.83)	11.57 (4.54)	11.76 (4.15)	11.24 (4.4)	10.98 (4.95)
EPS-25 Mean (SD)	4.23 (1.6)	4.00 (1.7)	4.17 (1.74)	3.63 (1.93)	3.56 (2.08)	3.59 (1.86)	3.31 (2.06)	3.2 (2.02)	3.11 (1.92)	3.02 (2.14)
IEE	3.02 (2.10)	2.65 (1.99)	2.89 (2.23)	2.50 (2.18)	2.26 (2.04)	2.31 (2.14)	2.41 (2.33)	2.08 (1.97)	2.16 (2.18)	1.85 (2.20)
AVO	3.81 (1.96)	3.84 (2.05)	4.26 (2.33)	3.48 (2.30)	3.49 (2.48)	3.32 (2.41)	3.10 (2.29)	2.94 (2.35)	3.15 (2.22)	2.97 (2.34)
UNE	5.57 (2.37)	5.34 (2.40)	5.42 (2.56)	4.44 (2.59)	4.69 (2.65)	4.98 (2.48)	4.27 (2.87)	4.47 (2.72)	3.89 (2.81)	3.71 (2.87)
SUP	4.97 (2.34)	4.72 (2.52)	4.86 (2.43)	4.65 (2.73)	4.04 (2.90)	4.25 (2.93)	3.80 (2.54)	3.58 (2.62)	3.75 (2.60)	3.83 (2.56)
UNG	3.78 (1.88)	3.47 (1.92)	3.40 (2.06)	3.06 (2.13)	3.32 (2.50)	3.10 (2.16)	2.96 (2.41)	2.93 (2.27)	2.60 (1.83)	2.75 (2.33)

*PHQ-15, Patient Health Questionnaire-15; EPS-25, Emotional processing scale-total; IEE, Impoverished emotional experience; AVO, Avoidance; UNE, Unprocessed emotions; SUP, Suppression; UNG, Unregulated Emotions.*

### Mediation Analysis

Table 2 shows the results from the single and multiple mediator analyses. The estimated average weekly change on the PHQ-15 was 0.29 (95% CI [0.21, 0.37]). The EPS-25 total score also changed significantly during treatment, with a slope of 0.13 (95% CI [0.10, 0.16]). In the single mediator analysis, all five subscales of the EPS-25 had statistically significant *ab*-products, indicating that change in each EPS-variable was associated with change in PHQ (Table 2, left column). In the multiple mediator analysis however, where the five potential mediators competed in explaining the change in somatic symptoms (PHQ-15), only Signs of Unprocessed Emotions, and Impoverished Emotional Experience subscales were significant (Table 2, right column). The total indirect effect (i.e., the sum of the *ab*-products for these two subscales in the multiple mediator model) was 0.15 [0.09, 0.24]. The proportion of the mediated effect was 0.49 (0.15/0.29), indicating that about half of the total effect on the PHQ-15 was accounted for by these two EPS-25 subscales.

**TABLE 2:** Indirect effects, *ab*-product, of the five tested mediators of the effect of treatment week on the primary outcome measure PHQ-15.

	Single mediator analysis		Multiple mediator analysis	
Mediator	*ab*	95% CI	*ab*	95% CI
EPS Impoverished Emotional Experience	0.09*	[0.05, 0.15]	0.054*	[0.03, 0.11]
EPS Signs of Unprocessed Emotion	0.11*	[0.06, 0.20]	0.068*	[0.03, 0.13]
EPS Avoidance	0.07*	[0.04, 0.13]	0.0016	[–0.04, 0.03]
EPS Suppression	0.07*	[0.04, 0.16]	0.012	[–0.01, 0.05]
EPS Unregulated Emotion	0.07*	[0.04, 0.15]	0.013	[–0.01, 0.05]
All mediators			0.15*	[0.09, 0.24]

*Statistical significance of indirect effects, ab-products, based on their respective bootstrapped 95% CIs not containing zero.

*EPS, Emotional Processing Scale—25 item version; PHQ-15, Patient Health Questionnaire-15.*

## Discussion

This study is one of the first attempts to examine changes in emotional processing in a short-term emotion-focused therapy, I-EAET. We found that a reduction in emotional processing difficulties—that is, an increased capacity for adaptive emotional processing—was closely related to a reduction in somatic symptoms in patients with somatic symptom disorder who were receiving a 9-week trial of I-EAET. Two facets or subscales of emotional processing were specifically and uniquely linked to reduced somatic symptoms: an increased capacity to be in contact with and aware of emotions (i.e., reduction in EPS-25 subscale Impoverished Emotional Experience) or not getting stuck or being overwhelmed by intrusive emotions or memories (EPS-25 subscale Signs of Unprocessed Emotions). This finding underscores the importance of certain emotional processes as potential vehicles of change.

The subscale Impoverished Emotional Experience overlaps with the construct alexithymia (Baker et al., 2010). Alexithymia, or difficulties identifying, describing, and sharing emotions, is known to be elevated in chronic pain conditions (e.g., migraine, fibromyalgia) and is positively associated with pain intensity and interference (Aaron et al., 2019). Alexithymia has long been considered difficult to treat (Sifneos, 1973; Ogrodniczuk et al., 2011) but recent studies show that it can be reduced (Cameron et al., 2014). Thus, the mediated effect of change in the subscale Impoverished emotional experience on somatic symptoms is both consistent with previous literature and plausible, given that EAET specifically aims to increase emotional awareness.

The EPS-25 subscale, Signs of Unprocessed Emotions, reflects emotions or traumatic memories that are not being processed properly but instead are intrusive and fragmented (Ehlers and Clark, 2000). Because EAET explicitly focuses on emotional exposure and fully processing emotions stemming from stressful life events, it is plausible that changes in this facet of emotional processing occurred during EAET. We propose that this finding is similar to that of treating post-traumatic stress disorder, which also can contribute to a reduction in somatic symptoms and disability (Gupta, 2013).

The weekly change in somatic symptoms and emotional processing was quite modest with PHQ-15 falling an average of 0.29 points per week, and EPS-25 dropping 0.13 points. However, these weekly reductions sum to yield rather substantial reductions over the course of 9 weeks of therapy. For example, the minimally clinically important difference (MCID) for the PHQ-15 score is a reduction of at least 2.3 points (Toussaint et al., 2017), whereas an increase of only 1 point is predicts a 3% increase in health care use (Toussaint et al., 2017).

One obvious limitation of this study is that it did not include a control or comparison condition, thereby rendering it difficult to attribute changes in somatic symptoms and emotional processing to the treatment rather than factors such as history or maturation. Randomized controlled trials are needed to obtain greater certainty and specificity. Second, the mediation analysis in this study can establish only a correlation between PHQ-15 and EPS-25 but precludes causal inferences. Although improved emotional processing could reduce symptoms, it also is possible that reduced symptoms permit better emotional processing. However, the weekly measurements of the outcome PHQ-15 during the treatment period provide an indication of *potential* treatment effect because it is likely that these weekly changes are to some extent associated with participation in treatment. In line with the same reasoning, weekly changes observed on the EPS-25 subscales during the treatment period are likely be associated with participation in treatment. As EAET aims to improve somatic symptoms by changing emotional processing, an association of change in EPS-25 and change in PHQ-15 as found in this study, is coherent and possibly in line with assumptions of EAET.

Taken together, this study gives preliminary evidence that improvements in emotional processing are related to reductions in somatic symptoms in an internet-administered EAET treatment for patients with centralized persistent physical symptoms.

## Data Availability Statement

The datasets presented in this article are not readily available because participants did not consent to this. Therefore, the dataset is available on reasonable requests as deemed by the principal investigator of the study. Requests to access the datasets should be directed to corresponding author.

## Ethics Statement

The studies involving human participants were reviewed and approved by the Swedish Ethical Review Authority (Dnr 2019-03317). The patients/participants provided their written informed consent to participate in this study.

## Author Contributions

DM and RJ designed the study with ML in an advisory role. DM wrote the first draft of the manuscript. BL did the statistical analysis. All authors contributed to revising the manuscript and accepting its final version.

## Conflict of Interest

BL was shareholder of Dahlia Qomit AB, a company specializing in online psychiatric symptom assessment, and Hedman-Lagerlöf och Ljótsson Psykologi AB, that licenses a treatment manual for irritable bowel syndrome on a commercial basis. HS was the owner of Mind Body Publishing, a company that sells books written by HS for patients dealing with mind body symptoms and for professionals who treat such patients. The remaining authors declare that the research was conducted in the absence of any commercial or financial relationships that could be construed as a potential conflict of interest.

## Publisher’s Note

All claims expressed in this article are solely those of the authors and do not necessarily represent those of their affiliated organizations, or those of the publisher, the editors and the reviewers. Any product that may be evaluated in this article, or claim that may be made by its manufacturer, is not guaranteed or endorsed by the publisher.
